# Efficacy and feasibility of vein of Marshall ethanol infusion during persistent atrial fibrillation ablation: A systematic review and meta‐analysis

**DOI:** 10.1002/clc.24178

**Published:** 2023-11-06

**Authors:** Wei‐Li Ge, Tao Li, Yi‐Fei Lu, Jian‐Jun Jiang, Tao‐Hsin Tung, Su‐Hua Yan

**Affiliations:** ^1^ Department of Cardiology Shandong Provincial Qianfoshan Hospital Shandong University Jinan Shandong China; ^2^ Department of Cardiology Taizhou Hospital of Zhejiang Province Affiliated to Wenzhou Medical University Zhejiang China; ^3^ Evidence‐Based Medicine Center, Taizhou Hospital of Zhejiang Province Wenzhou Medical University Linhai China; ^4^ Department of Urology Taizhou Hospital of Zhejiang Province Affiliated to Wenzhou Medical University, Enze Hospital, Taizhou Enze Medical Center (Group), Affilitated to Hangzhou Medical College Taizhou Zhejiang China; ^5^ Key Laboratory of Evidence‐Based Radiology of Taizhou Linhai Zhejiang China; ^6^ Department of Cardiology Shandong Provincial Qianfoshan Hospital Jinan China

**Keywords:** ablation, atrial fibrillation, atrial tachycardia, ethanol infusion, vein of Marshall

## Abstract

**Background:**

Catheter ablation (CA) is currently used to treat persistent atrial fibrillation (PeAF). However, its effectiveness is limited. This study aimed to estimate the effectiveness of the vein of Marshall absolute ethanol injection (VOM‐EI) for PeAF ablation.

**Hypothesis:**

Adjunctive vein of Marshall ethanol injection (VOM‐EI) strategies are more effective than conventional catheter ablation (CA) and have similar safety outcomes.

**Methods:**

We extensively searched the literature for studies evaluating the effectiveness and safety of VOM‐EI + CA compared with CA alone. The primary endpoint was the rate of acute bidirectional block of the isthmus of the mitral annulus (MIBB). The secondary endpoints were atrial fibrillation (AF) or atrial tachycardia (AT) recurrence over 30 seconds after a 3‐month blanking period. Weighted pooled risk ratios (RRs) and corresponding 95% confidence intervals (CIs) were calculated using a random effects model.

**Results:**

Based on the selection criteria, nine studies were included in this systematic review, including patients with AF (*n* = 2508), persistent AF (*n* = 1829), perimitral flutter (*n* = 103), and perimitral AT (*n* = 165). There were 1028 patients in the VOM‐EI + CA group and 1605 in the CA alone group. The VOM‐EI + CA group showed a lower rate of AF/AT relapse (RR = 0.70; 95% CI = 0.53–0.91; *p* = .008) and a higher rate of acute MIBB (RR = 1.29; 95% CI = 1.11–1.50; *p* = .0007) than the CA alone group.

**Conclusion:**

Our meta‐analysis revealed that adjunctive VOM‐EI strategies are more effective than conventional CA and have similar safety outcomes.

## Graphical abstract

Efficacy and feasibility of vein of Marshall ethanol infusion. Through literature search and meta‐analysis, it was found that adjunctive vein of Marshall ethanol injection (VOM‐EI) strategies are more effective than conventional catheter ablation (CA) and have similar safety outcomes.

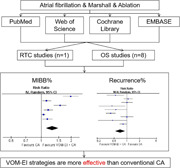

## 1 INTRODUCTION

During the past few decades, radiofrequency catheter ablation (CA) has become a widely accepted technique for treating atrial fibrillation (AF), particularly paroxysmal AF, with pulmonary vein isolation (PVI) as the standard strategy. However, the rate of freedom from multiple atrial arrhythmias 10 years after PVI for paroxysmal AF is approximately 62.7%, even lower in persistent atrial fibrillation (PeAF).^1^ For the latter, ablation of an additional line compartmentalizing the atria has been attempted as an additional strategy, given the high success rate in surgical maze procedures. However, it makes no additional contribution to treatment efficacy beyond PVI in randomized trials, mainly because of the difficulty in achieving durable lesions for bidirectional block.^2^, ^3^

The ligament of Marshall (LOM) consists of blood vessels (veins of Marshall [VOM]), muscle bundles, fibrous tissue, nerve fibers, ganglia, and fat, making it a source of triggers for atrial arrhythmias.^4^ The LOM has also been identified to play a critical role in residual conduction over the mitral isthmus (MI) and in left atrial (LA) reentrant tachycardias after AF ablation.^5^, ^6^, ^7^, ^8^ Moreover, CA alone cannot guarantee the effective destruction of the epicardial musculature of the LOM, which is mostly insulated by adipose tissue sheaths that act as insulating structures. To overcome these technical limitations, ethanol chemoablation can be performed by infusing ethanol into the VOM (VOM‐EI). This technique chemically damages the myocardium of the MI and epicardial fibers, contributing to mitral isthmus bidirectional block (MIBB) and durability.^9^, ^10^ Several single‐center reports and cases related to VOM‐EI have been published, among which only one randomized controlled trial (RCT) exists.^11^, ^12^, ^13^ In the present study, we systematically reviewed relevant publications and meta‐analyzed the effect size, focusing on the increase in block rate and improvement in long‐term rhythm control to update the knowledge and clinical evidence for the benefits of VOM‐EI.

## 2 METHODS

### 2.1 Data sources and retrieval strategy

We extensively searched relevant studies published before July 1, 2023, in the following databases: PubMed, Web of Science, Cochrane Library, and EMBASE, with the language restricted to English. Additional relevant studies were identified from the retrieved references. The search terms were as follows: (“chronic atrial fibrillation” or “persistent atrial fibrillation”), (“Vein of Marshall” or “Ligament of Marshall” or “Marshall vein” or “Marshall ligament”), (“Radiofrequency infusion” or “ablation”) (Supporting Information S2: Table 1).

### 2.2 Study selection

The present study conformed to the Preferred Reporting Items for Systematic Reviews and Meta‐Analyses (PRISMA) guidelines. The study protocol was prespecified in the International Prospective Register of Systematic Reviews (PROSPERO) network (PROSPERO ID: CRD42022339772). We selected RCTs and observational studies and excluded abstracts, animal studies, single‐arm studies, case series, editorials, reviews, case reports, and letters to the editor. Two investigators (W.‐L. G. and T. L.) independently searched and selected the studies. Discrepancies were resolved by a third investigator (T.‐H. T.).

### 2.3 Quality assessment

We assessed the quality of the included studies using the Revised Cochrane Risk‐of‐Bias Tool for Randomized Trials (RoB2) and the Newcastle–Ottawa Scale for observational studies. Two authors (W.‐L. G. and T. L.) independently assessed the bias in each study.

### 2.4 Data extraction

The data extracted from the final studies included the first author's last name, study design, publication year, follow‐up duration, country of origin, sample size, and efficacy endpoints (including MIBB and relapse of AF or atrial tachycardia [AT]). Finally, we focused on the following data: the number of patients who underwent VOM‐EI + CA or CA alone, their age, baseline comorbidities (including diabetes, stroke, hypertension, heart failure, and coronary artery disease), and characteristics before surgery (including LA diameter, left ventricular ejection fraction, and CHA2DS2‐VASc score).

### 2.5 Outcomes

The primary endpoint of our meta‐analysis was MI block after ablation. The secondary endpoint was the recurrence of any atrial arrhythmia of over 30 seconds after a 3‐month blanking period and periprocedural complications (pericardial effusion, stroke, or atrioesophageal fistula, etc.).

### 2.6 Statistical analysis

Meta‐analyses were performed using Review Manager version 5.3. A random effects model was used to calculate the weighted pooled risk ratios (RRs) and corresponding 95% confidence intervals. Statistical significance was set at *p* < .05. Heterogeneity was assessed using the Higgins *I* ^2^ index.^14^

## 3 RESULTS

### 3.1 Study selection

**Figure 1:**
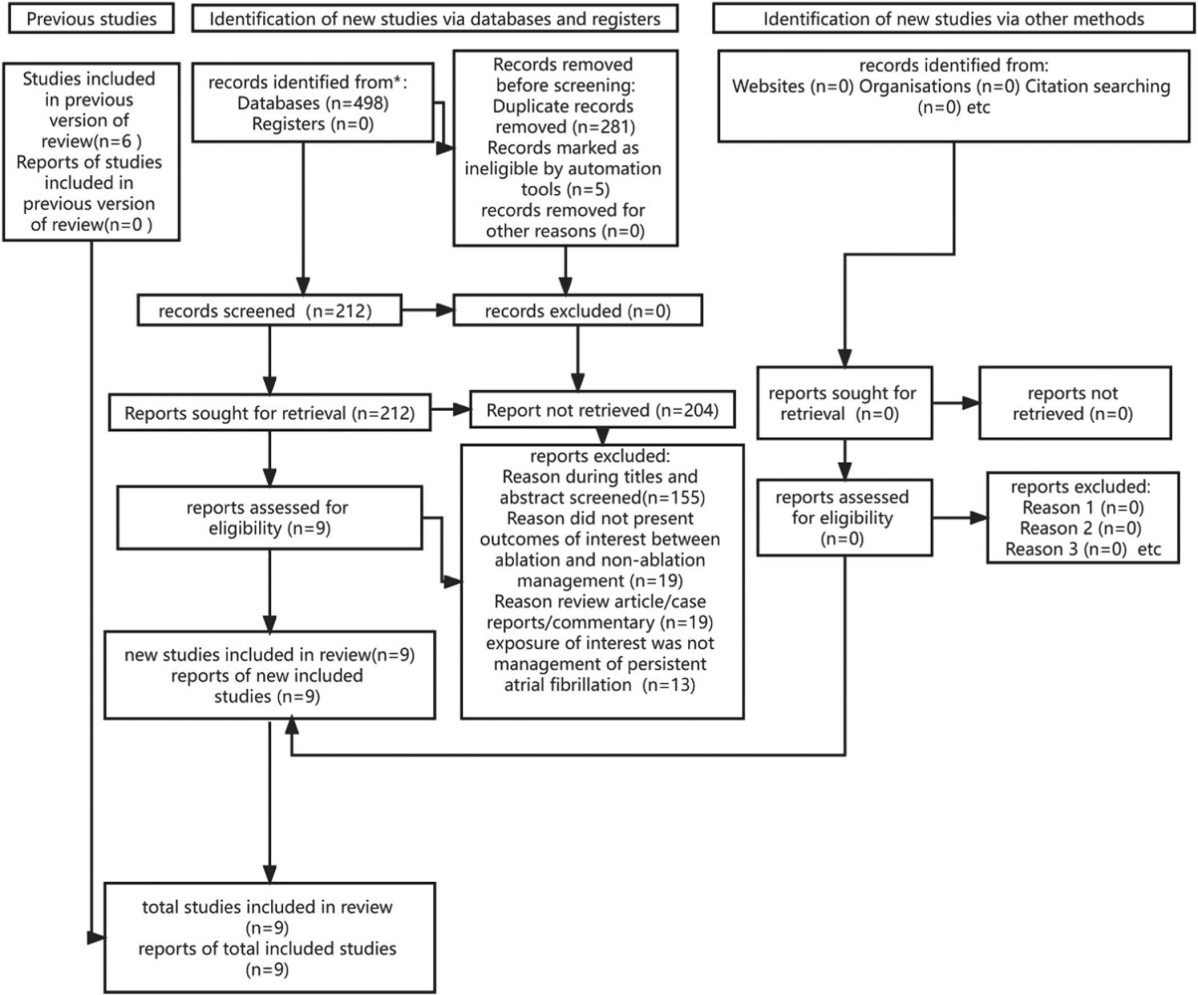
Preferred Reporting Items for Systematic Reviews and Meta‐Analyses flow diagram for the selection of studies.

In total, 498 studies were retrieved. Of these, 212 were included in this systematic review. Subsequently, 155 irrelevant articles with insufficient data were excluded based on their titles and abstracts. Finally, eight studies were retained. Figure 1 shows the PRISMA flowchart illustrating the study selection process.

### 3.2 Study characteristics

Table 1 lists the characteristics of the nine studies included in the meta‐analysis. A total of 2508 patients with AF were included in the study, of whom 1028 underwent VOM‐EI + CA, and 1605 underwent CA alone. The selected studies were published between 2019 and 2023. The study population included Europeans (*n* = 365), North Americans (*n* = 243), and East Asians (*n* = 1900). One of the studies was randomized controlled, and the others were observational. In these studies, 1852 (73.8%) participants were male; five enrolled mainly patients with persistent or nonparoxysmal AF, two enrolled patients with perimitral atrial tachycardia (PMAT), one enrolled a mix of patients with persistent and paroxysmal AF, and one enrolled only those with paroxysmal AF. There were some differences in baseline characteristics between the two groups. The prevalence of hypertension and diabetes was lower in the VOM‐EI + CA group. Moreover, patients in this group were older than those in the CA alone group. The other baseline characteristics were similar between the groups.

**Table 1:** summarizes the baseline comorbidities and preoperative characteristics, including LVEF, LA diameter, and CHA2DS2‐VASc score, among others.

Characteristics of studies included in the meta‐analysis																			
MIBB%	Study type	Country	AT subtype	Follow‐up duration, month	Efficacy endpoints	Safety end points	Male	Hypertension	Diabetes mellitus	CAD	Stroke	Heart failure	CHA2DS2 ± VA	LVEF, %	LA diameter	Effective%	References	VOM‐EI + CA group, *n*/CA alone	Age (years)
–	OS	Taiwan	Nonparoxysmal atrial fibrillation	12	Freedom from AF and arrhythmia recurrence	Periprocedural complications	29	7	5	6	5	4	1.7 ± 1.3	58.3 ± 4.2	42.3 ± 7.3	71.88%	Liu et al.^15^	32	56.4 ± 9.4
–							87	56	13	19	9	17	1.5 ± 1.2	57.4 ± 5.2	42.0 ± 4.5	30.20%		96	56.1 ± 10.2
87%	OS	Japan	Paroxysmal AF	12	Freedom from AF recurrence	Periprocedural complications	96	41	11	–	12	7	0.8 ± 0.8	70.0 ± 5.5	40.9 ± 3.4	71.21%	Okishige et al.^16^	132	63.2 ± 11.8
–							150	47	28	–	7	9	0.9 ± 0.7	63.4 ± 5.8	40.8 ± 2.9	71.43%		210	62.8 ± 11.1
96.80%	OS	France	Perimitral flutter	12	Acute MIBB block and AT recurrence	Decreasing complications	56	19	7	–	1	3	1.6 ± 0.8	54.7 ± 7.8	–	–	Takigawa et al.^4^	32	64.1 ± 8.5
88.70%							25	48	10	–	5	15	1.6 ± 2.3	55.3 ± 10.6	–	–		71	62.3 ± 7.6
97%	OS	Japan	Nonparosxysmal AF	12	MIBB block	–	138	117	49	0	–	29	1.6 ± 1.0	60.0 ± 9.8	48.5 ± 5.2	–	Ishimura et al.^17^	176	66.8 ± 8.8
92%							281	249	94	2	–	85	1.6 ± 0.9	60.0 ± 10.9	48.8 ± 6.1	–		384	67.6 ± 9.1
98.70%	OS	France	Persistent AF	12	Acute MI block and MIBB reconnection	Periprocedural complications	115	–	–	–	–	–	2.0 ± 1.5	57.1 ± 5.8	43.3 ± 13.5	–	Nakashima et al.^13^	152	63.8 ± 9.4
63.60%							90	–	–	–	–	–	2.0 ± 1.5	58.6 ± 10.5	40.4 ± 11.1	–		110	60.9 ± 9.2
95.50%	OS	China	Persistent AF	12	Freedom from AF/AT and all ablation lines block	Adverse event	47	32	11	12	6	19	–	58.7 ± 8.7	43.6 ± 5.5	87.90%	Lai et al.^18^	66	61.0 ± 10.9
80.80%							84	28	28	20	17	19	–	59.1 ± 7.7	42.7 ± 4.7	64.80%		125	61.1 ± 10.3
93.40%	OS	China	Perimitral atrial tachycardia	6	Acute MIBB block and AT recurrence	Periprocedural complications	52	48	9	10	7	8	1.9 ± 1.4	60.5 ± 7.3	42.0 ± 5.7	76.32%	Gao et al.^19^	76	61.5 ± 9.5
81%							51	52	17	14	11	14	2.3 ± 1.8	59.2 ± 7.7	43.0 ± 5.6	56.20%		89	63.1 ± 11.0
68.92%	OS	Japan	Nonparoxysmal/p aroxysmal AF	13	Freedom from AF/AT	–	134	110	53	0	–	–	1.9 ± 1.1	60.0 ± 10.2	49.0 ± 5.7	90.96%	Ishimura et al.^20^	177	69.0 ± 8.3
69.91%							156	156	61	1	–	–	1.8 ± 1.1	61.0 ± 8.9	48.0 ± 5.4	88.98%		236	69.0 ± 8.8
74.01%	RCT	The United States	Persistent AF	12	Freedom from AF	Facuteprocedural complications and total mortality	137	144	52	52	19	48	2.9 ± 1.6	52.1 ± 10.1	44.8 ± 7.9	49.20%	Valderrábano et al.^11^	185	66.6 ± 9.6
53.80%							124	104	31	41	19	42	2.6 ± 1.6	53.4 ± 9.4	47.0 ± 7.5	30%		158	66.4 ± 9.9

Abbreviations: AF, atrial fibrillation; AT, atrial tachycardia; CA, catheter ablation; CAD, coronary artery disease; LVEF, left ventricular ejection fraction; MIBB, mitral isthmus bidirectional block; OS, observational study; RCT, randomized controlled trial; VOM‐EI, vein of Marshall absolute ethanol injection.

### 3.3 Effect of VOM‐EI on MI ablation

**Figure 2:**
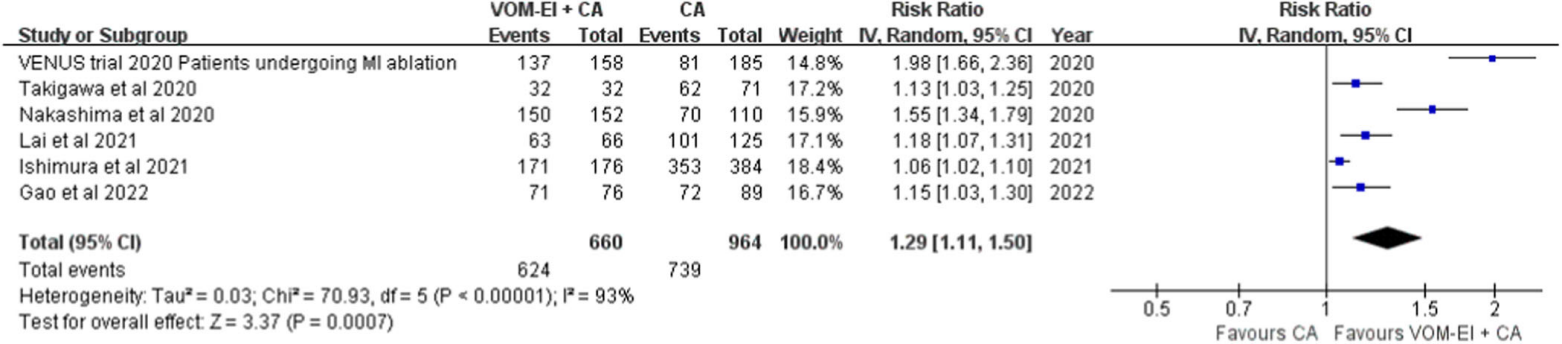
Pooled effect sized of VOM‐EI for mitral isthmus ablation. CA, catheter ablation; CI, confidence interval; VOM‐EI + CA, vein of Marshall absolute ethanol injection with catheter ablation.

Of all included studies, six reported procedural data for MI ablation; 624 (94.5%) of 660 patients undergoing VOM‐EI achieved MIBB, significantly higher than the 739 (82.7%) of 964 patients undergoing CA alone (RR = 1.29 [1.11–1.50], *I* ^2^ = 93%). The heterogeneity was largely due to the results of the study by Nakashima et al., who incorporated high‐density mapping to evaluate MIBB and found that only 63.6% of patients achieved MI block in the CA alone group (Figure 2).

### 3.4 Effect of VOM‐EI on recurrence

**Figure 3:**
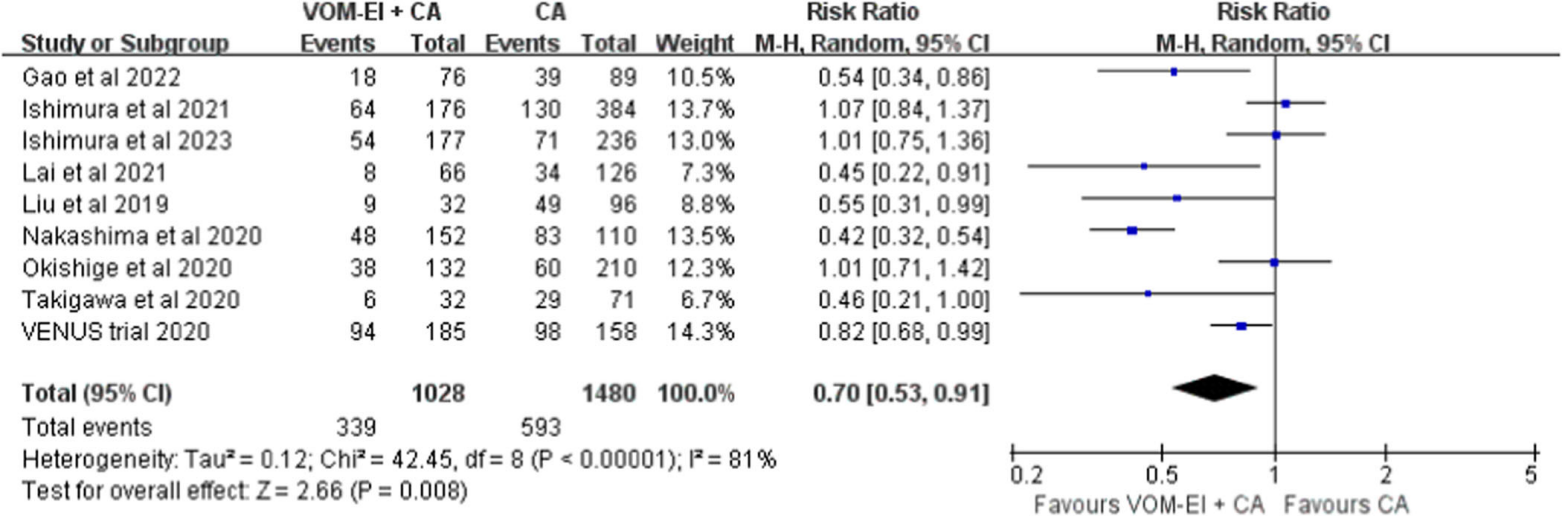
The overall pooled effect of VOM‐EI on recurrence of atrial arrhythmias. CA, catheter ablation; CI, confidence interval; M‐H, Mantel–Haenszel; VOM‐EI + CA, vein of Marshall absolute ethanol injection with catheter ablation.

Among all included studies, the randomized trial and five observational studies reported the benefit of VOM‐EI in rhythm control, while three studies found a neutral effect. The overall pooled effect size favored the benefit of VOM‐EI in reducing the recurrence of atrial arrhythmias (RR = 0.70 [0.53–0.91], *I* ^2^ = 81%; Figure 3). Considering that only persistent AF and PMAT are indicated for VOM‐EI in current clinical practice, a subgroup analysis of five studies with mainly PeAF or nonparoxysmal AF and two studies with only PMAT was performed, and no significant subgroup differences were identified (0.63 [0.42–0.95] vs. 0.52 [0.35–0.77], respectively, *p* = .49; Supporting Information S2: Figure 1). Furthermore, we compared the benefits of VOM‐EI in a subgroup of studies with and without MI ablation. Notably, in populations where MI ablation was not performed or MIBB was not achieved, VOM‐EI did not provide any rhythm control; however, in populations undergoing MI ablation or those who achieved MIBB, VOM‐EI was associated with a lower risk of recurrence (0.59 [0.42–0.82] vs. 0.96 [0.79–1.17], *p* = .01; Supporting Information S2: Figure 2).

### 3.5 Safety of VOM‐EI

After analyzing the complications, we found no significant differences in surgery risk with VOM‐EI (RR = 1 [0.67–1.48], *p* = .98; Supporting Information S2: Figure 3).

### 3.6 Quality assessment

The Newcastle–Ottawa Cohort Study Scale and the RoB2 were used to assess the quality of the selected studies. All the studies had low‐to‐moderate scores. In the Newcastle–Ottawa Scale System, each asterisk represents a single star. The largest asterisk is 2 for comparability and 1 for all other categories. All stars are counted in the total score. Scores of 5–6 indicated moderate quality and 7–9 indicated high quality. Funnel plots revealed no significant publication bias for MI block or recurrence of atrial arrhythmias (Supporting Information S2: Figure 4).

### 3.7 Quality assessment

We assessed the quality of the included studies by using the Newcastle‐Ottawa Scale for cohort studies and the Revised Cochrane risk‐of‐bias tool for RCTs, as shown in Supporting Information S2: Table 1 and Supporting Information S2: Figure 5. All studies scored low to moderate on the scales.

## 4 DISCUSSION

In this systematic review and meta‐analysis, we assessed publications related to VOM‐EI, including nine original research articles spanning more than a decade. The results revealed that VOM‐EI benefits MIBB and long‐term rhythm control. Furthermore, we compared the benefits of VOM‐EI in a subgroup of studies with and without MI ablation, we found that VOM‐EI was associated with a lower risk of recurrence.

The basic mechanisms of AF initiation and maintenance are complex, and AF ablation is still difficult because of its low success rates, increased risk of AT after AF ablation, and deterioration of atrial function. Despite the fact that a Randomized Controlled Multicenter Clinical Trial (STAR‐AF II) had shown that additional ablation, such as linear ablation and complex fractionated atrial electrogram, failed to reduce AF recurrence with long‐term follow‐up,^2^ it was nevertheless compared to PVI alone. However, linear lesions have been shown to be helpful in controlling rhythm during surgical ablation. According to theory, linear ablation aids in LA compartmentation, stops the rotors from circling the left atrium, and hence stops the maintenance of AF.^18^ The primary cause of this discrepancy is the failure of RF CA to result in increased SR maintenance because transmural lesions cannot always be obtained. Advancement of ablation technology or procedural, which can cause transmural lesions, might improve the benefits of linear ablation for PeAF ablation.

MI linear ablation is essential to CA with persistent AF. However, CA is difficult to achieve transmural lesion due to the thickness of the atrial wall, for example, in the left lateral ridge which is both part of MI and insertion of epicardial connection used separately to complete MI linear ablation, resulting in a reduced rate of MI block because of the epicardial conduction of the VOM. The success rate of MI blocks is between 51% and 68%,^12^ and most patients require additional ablation within the coronary sinus (CS). Multiple Marshall bundle (MB)‐LA/CS connections further decrease the probability of achieving MIBB.^7^ Acute conduction recovery in the MB is common in patients undergoing MI linear CA. It is difficult to create MI blocks, and the possibility of PMAT development is increased by an incomplete or disconnected MI line.^21^ PMAT has a significant recurrence rate (up to 25.6%–57.7%).^22^, ^23^ VOM‐EI could offer a way to accomplish MIBB. Ethanol can infiltrate into the atrial myocardium through the VOM and its collateral flow and quickly create transmural lesions that mostly affect the posterolateral LA free wall and anterior half of the left pulmonary antrum.^24^ More importantly, it has been demonstrated that lesions caused by ethanol infusion are more durable, which precludes subsequent reconnection across the blocked MI line.^13^ A growing amount of research has shown that VOM is significantly associated with arrhythmias and possible pathophysiological links between the condition and atrial arrhythmia have also been established. These include proximal VOM‐related AT, reentrant activities related to VOM that cause AF, focal activities that induce AF, and an unbalanced autonomic nervous system. VOM‐EI helps eliminate AF triggers^9^ and regional innervation, as well as facilitates^10^ the achievement of MIBB.^11^

Combining VOM‐EI with CA increased the rate of MIBB achievement and improved the maintenance time of sinus rhythm after AF ablation. Our meta‐analysis revealed that MIBB was achieved in 82.7% of the patients with AF who underwent VOM‐EI with CA. This technique improved MIBB achievement in patients with AF compared with CA alone. The advantages of VOM‐EI have been proven in other outcomes, such as relapse of PMAT. Our meta‐analysis revealed that VOM‐EI + CA has an overall reduction in the recurrence of atrial arrhythmias. Moreover, VOM‐EI with CA reduced the risk of AF and AT relapse by 10% in patients with PeAF compared with CA alone. Moreover, a large RCT indicated that in patients with PeAF, adjuvant VOM‐EI significantly enhanced the probability of being free from AF or AT 6 and 12 months postprocedure compared with CA alone.^12^ Additionally, CS ablation may increase the risk of tamponade, vessel injury, steam pop, and other complications. After analyzing the complications, we found no significant differences in surgery risk with VOM‐EI. Moreover, VOM‐EI is safe and attainable for treating injured regional tissue of the left atrium and improving parasympathetic denervation. Our meta‐analysis revealed that VOM‐EI + CA is a safe and effective option for PeAF and PMAT by increased rate of MIBB compared with CA alone.

### 4.1 Study limitations

Our study had a few limitations. First, as in other meta‐analyses, a publication bias may have affected our results. Publication bias was diminished as much as possible through a massive investigation of public documents. In addition, we performed created funnel plots and conducted Egger test analysis, although their effectiveness was limited because the number of selected studies was not sufficiently large. Most included studies were not randomized and had selection biases despite the comparatively high‐quality assessment scores. Second, different studies used different criteria for identifying relapse, such as an AF/AT duration > 30 seconds in some studies and >1 minute in others. Additionally, different methods of rhythm monitoring, such as continuous, event, and 24‐hour Holter monitoring, were used. Most of the included studies used 24‐hour Holter monitoring, which may not have detected all cases of AF recurrence. Moreover, the duration of follow‐up varied from 6 to 46.8 months, with four studies having follow‐up durations of <12 months. Finally, the number of studies included was limited due to the lack of relevant published data, which may have made the summary statistics insufficiently accurate. Although the present meta‐analysis included all the studies we could identify, there is still doubt regarding the external validity of these results owing to the small sample size. Therefore, further research should be conducted to verify the findings of this study and demonstrate whether VOM‐EI is effective in other procedures.

## 5 CONCLUSION

Our meta‐analysis demonstrated that VOM‐EI + CA has superior efficacy compared with CA alone in patients with AF with long‐term follow‐up. The VOM‐EI + CA group significantly improved from AF/AT and MIBB blocks. From a clinical perspective, adjunctive VOM‐EI + CA facilitates MI block and reduces the recurrence rate after ablation in patients with PeAF, deserves more clinical attempts and applications. More RCTs with larger cohorts and longer follow‐up periods are required to clarify the clinical outcomes.

## AUTHOR CONTRIBUTIONS

Wei‐Li Ge is the primary investigator and drafted the protocols, while Tao Li, Yi‐Fei Lu, and Jian‐Jun Jiang commented on and edited these. Tao Li carried out the title/abstract screening. Yi‐Fei Lu and Jian‐Jun Jiang carried out full‐text screening and the data‐extraction pilot. The corresponding author (Tao‐Hsin Tung) attests that all listed authors meet authorship criteria and that no others meeting the criteria have been omitted. The corresponding author (Su‐Hua Yan) accepts full responsibility for the finished work and the conduct of the study, has access to the data, and controls the decision to publish.

## CONFLICT OF INTEREST STATEMENT

The authors declare no conflict of interest.

## Supporting information

Supporting information.

## Data Availability

Data is available on reasonable request from the corresponding author.
