# Supplementary material for: Efficacy and feasibility of vein of Marshall ethanol infusion during persistent atrial fibrillation ablation: A systematic review and meta‐analysis
Source: Clin Cardiol. 2023 Nov 6;47(1):e24178. doi: 10.1002/clc.24178 (PMC10766122; doi:10.1002/clc.24178)
Supplement: Supplementary file 1 — Supporting information. [file CLC-47-e24178-s001.docx]

**Supplement Table 1.** The Newcastle-Ottawa Scale for assessing the quality of nonrandomized studies in meta-analysis

| **Table****:** The Newcastle-Ottawa Scale for assessing the quality of nonrandomized studies in meta-analysis | **Outcome** | **Total score** | 6 | 7 | 7 | 7 | 7 | 7 | 7 | 7 |
| --- | --- | --- | --- | --- | --- | --- | --- | --- | --- | --- |
|  |  | **Adequate follow-up** | * | * | * | * | * | * | * | * |
|  |  | **Enough follow up duration** | * | * | * | * | * | * | * | * |
|  |  | **Assessment of outcome** | * | * | * | * | * | * | * | * |
|  | **Selection** | **Comparability of the cohort** | * | * | * | * | * | * | * | * |
|  |  | **Outcome not present at baseline** | - | - | - | - | - | - | - | - |
|  |  | **Ascertainment of exposure** | * | * | * | * | * | * | * | * |
|  |  | **Selection of the non-exposed cohort** | - | * | * | * | * | * | * | * |
|  |  | **Representative ness of the exposed cohort** | * | * | * | * | * | * | * | * |
|  |  | **Study** | Liu et al,2019[15] | Nakashima et al,2020[14] | Takigawa et al，2020[17] | Ishimura et al，2021[18] | Okishige1 et al，2020[16] | Lai et al,2021[19] | Gao et al,2022[20] | Ishimura et al，2023[21] |


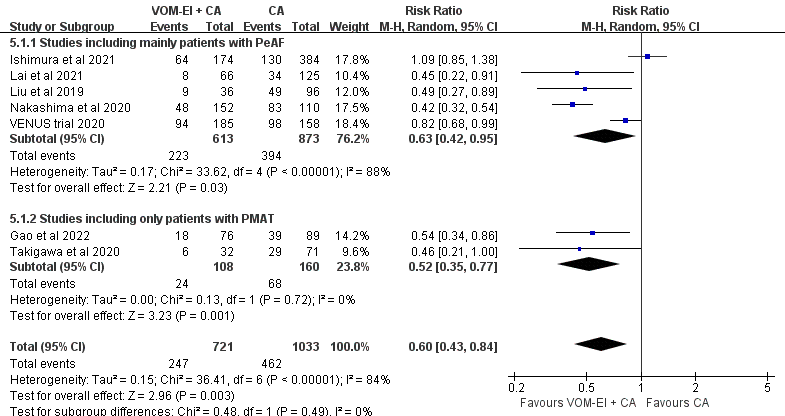


**Supplement Figure 1.** Subgroup analysis for studies including mainly patients with PeAF and studies including only patients with PMAT.(PeAF, Persistent atrial fibrillation; PMAT, perimitral atrial tachycardia; VOM-EI + CA, VOM absolute ethanol injection with catheter ablation; CA, catheter ablation)


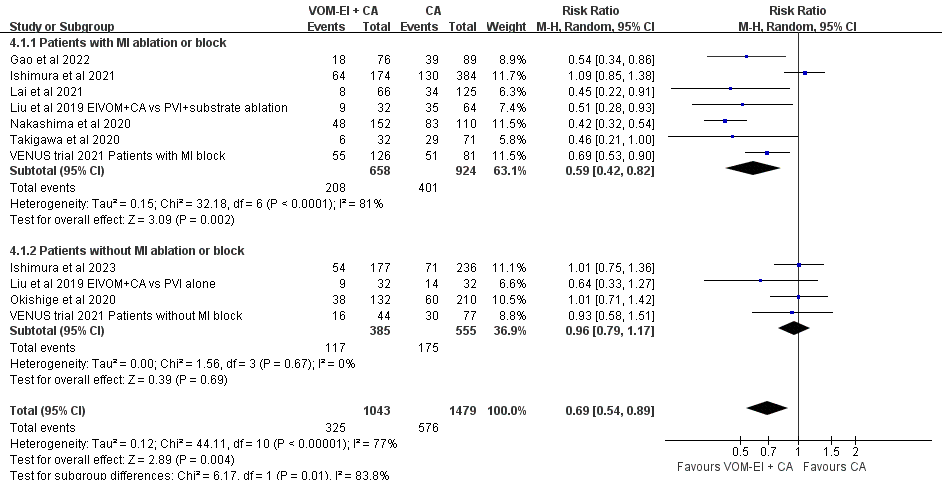


**Supplement Figure 2.** Subgroup comparison between population undergoing MIBB ablation or with MIBB block and population without MIBB ablation or block.(MIBB, mitral isthmus bidirectional block; VOM-EI + CA, VOM absolute ethanol injection with catheter ablation; CA, catheter ablation)

**
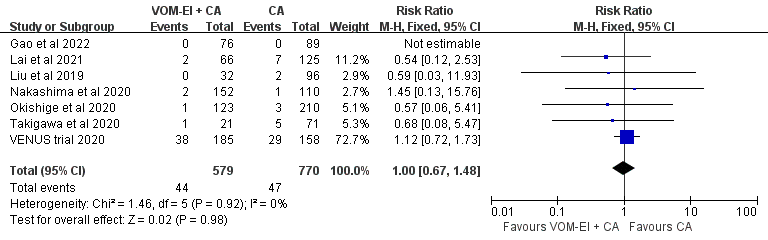
**

**Supplement Figure 3.** Pooled safety of VOM-EI and CA.(VOM-EI + CA, VOM absolute ethanol injection with catheter ablation; CA, catheter ablation, safety endpoint including intraprocedural pericardial effusion, subacute pericardial effusion requiring drainage, subacute pericardial effusion/pericarditis not requiring drainage, vascular access complications, stroke, transient ischemic attack, fluid overload, pneumonia atrioesophageal fistula and death)


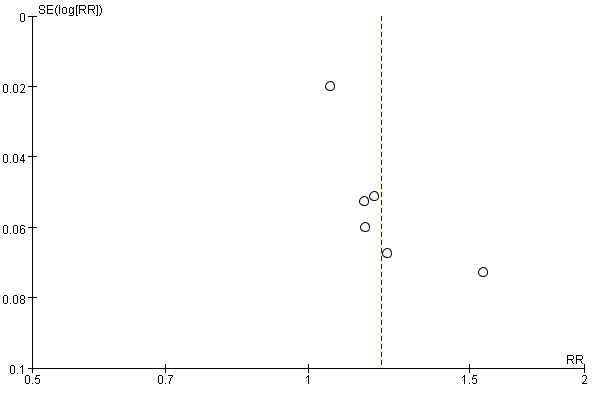

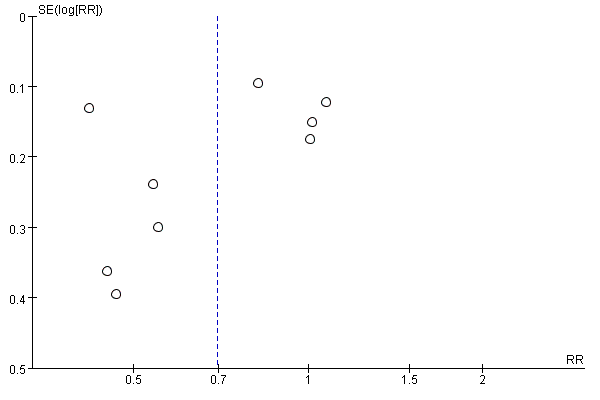


**Supplement Figure 4.** Funnel plot for studies investigating MIBB block (left panel) and recurrence of atrial arrhythmias (right panel).(MIBB, mitral isthmus bidirectional block)


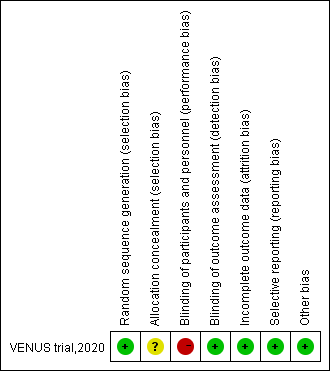


**Supplement Figure 5.** Quality assessment for randomized clinical trials according to the Cochrane risk of bias assessment tool.
